# Mechanisms of viability loss in dehydrated *Metarhizium anisopliae* blastospores

**DOI:** 10.3389/fmicb.2026.1764301

**Published:** 2026-05-07

**Authors:** Natasha Sant Anna Iwanicki, Gilberto Úbida Leite Braga, Guilherme Thomaz Pereira Brancini, Carem Gledes Vargas Rechia, João Paulo Rodrigues Marques, Elzbieta Stepula, Anant V. Patel, Italo Delalibera Júnior

**Affiliations:** 1 Department of Entomology and Acarology, “Luiz de Queiroz” College of Agriculture (ESALQ), University of São Paulo (USP), Piracicaba, SP, Brazil; 2 Department of Clinical, Toxicological and Bromatological Analyses, School of Pharmaceutical Sciences of Ribeirão Preto, University of São Paulo, Ribeirão Preto, SP, Brazil; 3 Department of BioMolecular Sciences, School of Pharmaceutical Sciences of Ribeirão Preto, University of São Paulo, Ribeirão Preto, SP, Brazil; 4 Department of Basic Sciences, School of Animal Science and Food Engineering, University of São Paulo, Pirassununga, SP, Brazil; 5 Bielefeld Institute of Applied Materials Research, Fermentation and Formulations of Biologicals and Chemicals, Hochschule Bielefeld - University of Applied Sciences and Arts (HSBI), Bielefeld, Germany

**Keywords:** entomopathogenic fungi, lipid peroxidation, oxidative stress, primary metabolites, Raman spectroscopy

## Abstract

The commercial use of *Metarhizium anisopliae* blastospores is significantly limited by their poor storage stability, mainly due to viability loss over time. Understanding the mechanisms underlying this decline is therefore essential to improve blastospore-based products. In this study, we investigated factors associated with viability loss in blastospores dried to two water activity levels, a_w_ = 0.1 (BS01) and a_w_ = 0.3 (BS03), representing more severely and moderately desiccated states, respectively. Samples were stored at 28 °C for 90 days and analyzed every 30 days using viability assays, flow cytometry, protein oxidation analysis, GC–MS, HPLC, and Raman spectroscopy. Viability declined progressively by approximately three orders of magnitude during storage, with BS01 showing a markedly faster reduction than BS03 during the first 30 days. Early storage stages were characterized by high oxidative stress, including elevated reactive oxygen species, lipid peroxidation, and impaired membrane integrity, all of which declined over time as metabolic activity decreased. In contrast, protein carbonylation increased continuously, reaching 11.02 ± 2.23 nmol/mg protein after 90 days. Metabolic analysis of BS01 revealed disrupted cellular homeostasis, including trehalose depletion and glucose accumulation, suggesting impaired metabolic capacity. Raman spectroscopy confirmed progressive molecular alterations in proteins, lipids, and polysaccharides. Together, these findings indicate that cumulative oxidative damage, particularly irreversible protein carbonylation, combined with progressive metabolic dysfunction, is a central driver of blastospore viability loss during storage.

## Graphical abstract

**Figure:**
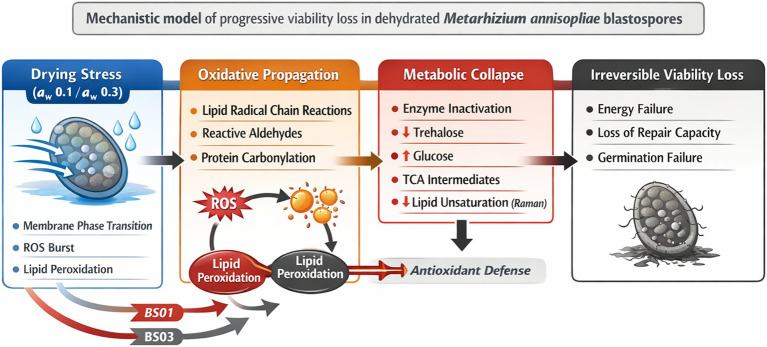

## 1 Introduction

The genus *Metarhizium* (Hypocreales: Clavicipitaceae) comprises a noteworthy group of entomopathogenic fungi within the Ascomycota, with as many as 11 species currently used worldwide for biological pest control (Zimmermann, 2007; van Lenteren et al., 2017). Various species of *Metarhizium* exhibit a versatile ecological lifestyle, enabling them to inhabit soil (Kepler et al., 2015; Rezende et al., 2015; Iwanicki et al., 2019; Botelho et al., 2019), infect insects and mites (Vega et al., 2009; Zimmermann, 2007), associate with plant roots, or exist as endophytes (Wyrebek et al., 2011; Sasan and Bidochka, 2013; Brancini et al., 2022; Panwar and Szczepaniec, 2024). In Brazil, there are currently 123 *Metarhizium*-based plant protection products registered (Iwanicki and Delalibera, 2025). One of the most notable examples of the successful application of *Metarhizium* is the large-scale aerial spraying of *Metarhizium anisopliae* conidia in sugarcane fields in Brazil (Iwanicki et al., 2019; Parra, 2014), which has become one of the most successful biological control programs worldwide.

Recent advances can shift production from traditional solid-state fermentation toward liquid culture fermentation, which enables the generation of different fungal propagules for integrated pest management, including blastospores (Iwanicki et al., 2021; Jaronski and Mascarin, 2017; Lane et al., 1991; Kleespies and Zimmermann, 1992), submerged conidia (Thomas et al., 1987; Jaronski and Mascarin, 2017; Iwanicki et al., 2021), and microsclerotia (Jackson and Jaronski, 2009; Kobori et al., 2015). M*etarhizium* blastospores can be rapidly produced in submerged culture (2–3 days) and are infective to various pests (Jaronski and Mascarin, 2017; Alkhaibari et al., 2016; Iwanicki et al., 2019, 2020a, 2020b, 2021). Although liquid fermentation offers operational advantages, the commercial application of blastospores remains limited by their lower tolerance to desiccation and storage stress than that of aerial conidia. Despite advances in achieving high yields of *Metarhizium* blastospores in low-cost media and in developing wettable powder formulations (Iwanicki et al., 2020a, 2020b, 2021), their commercial application remains limited by short shelf life.

One strategy to extend the shelf life of cells, such as blastospores, is to reduce primary metabolism using drying processes. Cells can be dried using various methodologies, such as spray drying, air drying, and lyophilization. Additionally, drying can occur while cells are embedded in various formulations and at different moisture levels or water activities. Water activity (a_w_) is the preferred parameter over moisture content because it reflects the amount of water available within cells for enzymatic reactions (ranging from 0 to 1), while moisture content only expresses the total quantity or its interaction with matrix components (Prior, 1979). Studies with aerial conidia of *Trichoderma* and *Beauveria* dried to a water activity a_w_ < 0.1 have shown that conidial viability can be maintained for prolonged periods, ranging from several months to more than two years, depending on storage temperature and packaging conditions (de Faria et al., 2022). Based on our experience, drying to an a_w_ ~ 0.1–0.2 extended the viability of the aerial conidia of entomopathogenic fungi.

During the drying process, blastospores undergo osmotic and oxidative stress (Rocca et al., 2019; Manzanera, 2021). Dietsch et al. (2021) summarized the intracellular effects that potentially occur during the desiccation of blastospores, including damage to the plasma membrane, proteome, and DNA. For instance, blastospores may experience alterations in the fluidity of their plasma membranes, affecting molecular diffusion, the production of reactive oxygen species that directly impact lipid peroxidation, and the oxidation of proteins and nucleic acids. Not only can these processes occur during drying and storage, but numerous molecular damages are also induced upon rehydration, leading to cell death (Marks et al., 2025; Kieliszek, 2025). Several studies have evaluated the shelf-life of entomopathogenic fungal blastospores under different formulations and storage conditions—including modified atmospheres, freezing (−20 °C), refrigeration (4–6 °C), and temperatures between 25 and 30 °C (Lane et al., 1991; Jackson and Jaronski, 2009; Mascarin et al., 2016; Iwanicki et al., 2021; de Faria et al., 2022; Lima et al., 2024). Previous investigations mainly investigated viability over time, but none have systematically explored the biochemical and molecular causes of blastospore viability loss during storage. This study fills that gap by linking cellular and molecular stress responses to viability decline and offering a framework for enhancing blastospore stability and shelf-life.

Here, we employed a multidisciplinary approach, combining biochemical assays, sugar and polyol profiling, metabolomics, and Raman spectroscopy to investigate the primary mechanisms underlying viability loss in *M. anisopliae* blastospores exposed to two water-activity levels (a_w_ = 0.1 for BS01; a_w_ = 0.3 for BS03), representing moderate and severe desiccation. Following the desiccation, the samples were stored at 28 ± 2 °C for up to 90 days. To identify potential primary metabolites, we analyzed the metabolome of both fresh and dried blastospores down to a water activity a_w_ = 0.1. Our hypotheses were as follows: (1) Poor storage stability of *M. anisopliae* blastospores is linked to one or a combination of the following parameters: increased protein carbonylation, lipid peroxidation, accumulation of reactive oxygen species (ROS), and significant damage to the plasma membrane; (2) the extent of plasma membrane damage, ROS accumulation and lipid peroxidation in *M. anisopliae* blastospores during storage depend on final water activity; and (3) xeroprotectants like sugars, amino acids, and polyols may accumulate under extreme drying.

## 2 Materials and methods

### 2.1 Isolate selection

The *Metarhizium anisopliae* strain ESALQ1184 was selected based on prior findings (Iwanicki et al., 2019), which show that it produced 1.2 × 10^8^ blastospores mL^−1^ when cultured in modified Adamek’s medium supplemented with minerals and vitamins. The medium composition per liter consisted of 80 g of yeast extract, 40 g of corn steep liquor, and 140 g of glucose. The mineral solution contained 2.5 g of potassium dihydrogen phosphate (KH₂PO₄), 1.0 g of calcium chloride dihydrate (CaCl₂·2H₂O), 0.83 g of magnesium sulfate heptahydrate (MgSO₄·7H₂O), 0.3 g of ferrous sulfate heptahydrate (FeSO₄·7H₂O), 29.6 mg of cobalt chloride hexahydrate (CoCl₂·6H₂O), 12.8 mg of manganese sulfate monohydrate (MnSO₄·H₂O), and 11.2 mg of zinc sulfate heptahydrate (ZnSO₄·7H₂O). The vitamin solution included 0.2 mg each of thiamine, riboflavin, calcium pantothenate, niacin, pyridoxamine, and thioctic acid, as well as 0.02 mg each of folic acid, biotin, and vitamin B₁₂. Sterile-filtered vitamin and trace element solutions were added after autoclaving, and the final pH was adjusted to 6.8. The ESALQ1184 strain is deposited in the Entomopathogenic Fungal Collection at ESALQ-University of São Paulo (Piracicaba, Brazil). A stock culture of conidia was obtained from sporulated cultures grown on dextrose agar (PDA, Difco^®^, Sparks, MD, USA), and preserved as sporulated agar chunks immersed in sterile 10% glycerol solution at −80 °C.

### 2.2 Fungal cultivation and drying procedure

The strain ESALQ1184 was grown in 50 mL liquid medium in two 250 mL baffled Erlenmeyer flasks (Bellco^®^ Glass, Vineland, NJ, USA) with two replicates. The fungus was first grown for 3 days in a preculture medium composed by 40 g L^−1^ glucose (Exodo científica^®^, Sumaré, SP, Brazil), 40 g L^−1^ yeast extract (Synth^®^, Piracicaba, SP, Brazil), mineral salts, trace metals, and vitamins adapted from Jackson’s medium (Jackson et al., 1997) at the following concentrations per liter: KH_2_PO_4_, 2.5 g; CaCl_2_·2H_2_O, 1.0 g; MgSO_4_·7H_2_O, 0.83 g; FeSO_4_·7H_2_O, 0.3 g; CoCl_2_·6H_2_O, 29.6 mg; MnSO_4_·H2O, 12.8 mg; ZnSO_4_·7H_2_O, 11.2 mg with a carbon-to-nitrogen (C: N) ratio of 12:1 (Iwanicki et al., 2021). The preculture medium was inoculated with 5 mL of a suspension containing 5 × 10^6^ conidia mL^−1^ and had a working volume of 50 mL. Blastospores produced in preculture were used to inoculate culture medium with the same composition but with 80 g L^−1^ glucose (Exodo científica^®^, Sumaré, SP, Brazil). A volume of 3.6 L was inoculated in a 6 L stirred bioreactor Minofors 2 (Infors HT^®^, Bottmingen, Switzerland) with 400 mL of suspension containing 5 × 10^7^ blastospores mL^− 1^. The initial pH was adjusted to 6.8 before autoclavation, and the temperature was maintained at 28 ± 2 °C. The gas flow was purged with filtered atmospheric air at a rate of 1.5 L min^− 1^, and the agitation speed was set to 400 rpm. During culture days, the agitation speed was increased to 600 rpm to maintain a dissolved oxygen level of at least 20%. Blastospores were cultured for 72 h.

After cultivation, the broth was subjected to vacuum filtration using a Büchner funnel fitted with 8 cm diameter Whatman filter paper (10–12 μm pore size) to retain the mycelial biomass. This pore size was selected to effectively separate hyphal fragments from unicellular blastospores based on their size differences. The resulting filtrate was examined under a microscope to confirm the absence of hyphal fragments and to ensure blastospore purity. The blastospores were then collected after centrifugation at 6000 rpm and 10 °C and washed three times with potassium-buffered saline (PBS) (8 g L^−1^ NaCl, 0.2 g L^−1^ KCl, 1.44 g L^−1^ Na₂HPO₄, and 0.24 g L^−1^ KH₂PO₄, pH 7.4) to remove residual culture medium. Following the final wash, the cells underwent an additional centrifugation process, allowing for the collection of the biomass, which was subsequently dried in an airflow chamber with continuous air circulation, but without temperature control. After a 24 h drying interval, the biomass was placed in an electrical blender, and short pulses of 2 s were applied to break it down into smaller particles. Next, the mixture was put into aluminum trays for drying in a controlled air-dry chamber, and the water activity was monitored every 2 h using a water activity meter (Novasina^®^–Labmaster AW, Lachen, Switzerland). Upon achieving an a_w_ = 0.30, half of the biomass was harvested from the chamber, portioned into aluminized moisture-proof and oxygen-impermeable bags, and vacuum-packed at a temperature of 28 ± 2 °C. The drying process of the residual biomass continued until it reached an a_w_ = 0.1, at which point it was portioned and stored at 28 ± 2 °C. The drying time, which decreased a_w_ = 0.3 to a_w_ = 0.1, varied from 7 h in the first experiment to 9 h in the second. Viability analyses were performed on fresh cells (before drying) and those stored for 7, 30, 60, and 90 days using the colony-forming unit (CFU) method.

### 2.3 Evaluation of membrane damage, ROS generation, and lipid peroxidation in blastospores under desiccation

Blastospore suspensions at a concentration of 10^7^ blastospores mL^−1^ were prepared from fresh and dried blastospores with water activity a_w_ = 0.1 (BS01) and a_w_ = 0.3 (BS03) samples before treatment with fluorophores. Blastospore membrane integrity was evaluated using flow cytometry analysis of cells stained with propidium iodide (PI) (ThermoFisher^®^, Waltham, MA, USA). At the same time, the overall levels of reactive oxygen species (ROS) were quantified using the ROS^®^ assay kit (ThermoFisher^®^, Waltham, MA, USA) according to the manufacturer’s specifications. After rehydration, samples were incubated with the ROS probe for 60 min, as recommended by the manufacturer, prior to cytometric analysis. This incubation period was standardized for all samples, treatments, and storage times to ensure consistency in ROS detection. Lipid peroxidation was assessed using the Image-iT Lipid Peroxidation Kit (ThermoFisher^®^, Waltham, MA, USA) according to the manufacturer’s guidelines. This kit utilizes the BODIPY™ 581/591 C11 undecanoic acid, a fluorescent probe sensitive to lipid peroxidation for assays conducted across various cell types. The fluorescence signal in the cells shifted from red to green in response to lipid peroxidation. By employing Texas Red^®^ (590 nm) and FITC (510 nm) emission filters, lipid peroxidation was quantified through the measurement of fluorescence intensities and the ratio of intensity within the Texas Red^®^ channel to the intensity present in the FITC channel. The greater this ratio, the lesser the lipid peroxidation observed in the sample. Because the lipid peroxidation assay requires rehydrated cells and fluorescence-based detection, the obtained signal may reflect the combined effects of drying, storage, and rehydration. For this reason, all samples were subjected to the same standardized rehydration and staining procedure to ensure comparability among treatments. Propidium iodide staining was performed using a 3 μM working solution, prepared by diluting the 1 mg mL^−1^ (1.5 mM) stock solution 1:500 in distilled water. Samples were incubated with PI for 10 min at room temperature in the dark prior to acquisition and according to the guidelines provided for the lipid peroxidation and reactive oxygen species assay kits. Cytometric analysis was conducted using a BD FACSCanto I flow cytometer, supplemented by BD FACSDiva software (BD Biosciences). For each sample, a total of 20,000 events were scrutinized, resulting in a comprehensive analysis of 40,000 events per fluorophore and per sample, considering two experiments. The fluorescence threshold (cut-off) was established based on unstained controls to determine background autofluorescence and define the positive staining gate. The two independent experiments were conducted as fully replicated biological experiments. In each experiment, fungal cultivation, blastospore production, and the subsequent drying process were performed independently, starting from separate inocula and culture batches.

The same dried blastospore samples used for flow cytometry and biochemical marker analyses were also used for viability determination throughout storage, allowing direct comparison between physiological and biochemical parameters across the same experimental material.

### 2.4 Protein carbonyl assay

For each storage time, 5 mg of BS01 was transferred to 2 mL microtubes, and 1 mL of RIPA Buffer (Merck^®^, Darmstadt, HE, Germany) was added. A screw cap with 1 mm zirconia-silica beads (BioSpec^®^, Bartlesville, OK, USA) was used. The microtubes were sealed and placed on a high-efficiency bead mill homogenizer (L-Beader 24, Loccus Biotechnology, Cotia, SP, Brazil) for four cycles of 30 s at a speed of 4.5 m s^−1^. After homogenization and cell lysis, the tubes were immediately placed on a cold rack, and the resultant lysate was collected and transferred to a sterile 1.5 mL Eppendorf tube. The lysate was then centrifuged at 10,000 g for 15 min at 4 °C. The supernatant was then transferred to a separate, clean tube, placed on the cold rack, and analyzed for absorbance at 280 nm and 260 nm to determine the presence of any contaminating nucleic acids within the sample. Then, protein carbonylation was evaluated using the Protein Carbonyl Colorimetric Assay (Cayman^®^, Ann Arbor, MI, USA), which employs the 2,4-Dinitrophenylhydrazine (DNPH) reaction to quantify protein carbonyl levels. The quantity of protein-hydrozone generated was assessed spectrophotometrically at a wavelength of 370 nm. Subsequently, the carbonyl content was normalized to the protein concentration determined from the final pellet using the BCA Protein Assay Kit (Merck, Darmstadt, Germany). Protein carbonylation assays were performed exclusively for the BS01 treatment due to resource limitations. BS01 was chosen as it had the largest initial viability drop and greater dehydration stress, making it most relevant for studying oxidative damage.

### 2.5 Fluorescence microscopy

The slides of sample BS01 and their subsequent suspension in an aqueous medium were analyzed with a Leica DM 6000 microscope using the follow filter: To detect fungal cell wall we used calcofluor white and analyzed under A4 Filter–ex: 340–380 nm; em: 450–490 nm. To detect lipid per oxidation we used an Image-iT^®^ Lipid Peroxidation Kit following the manufacturer instructions. C11B fluorescence was excited with an LED light source. The fluorophore works in two ways, when reduced Lipid Peroxidation Sensor is 581/591 nm analyzed under Y3 and when oxidized, the probe shifts the excitation and emission to 488/510 nm analyses under GFP filter. C11B images were captured at room temperature with a 10 × − 63×, plan-apochromat lens.

### 2.6 Scanning electron microscopy

Scanning electron microscopy of blastospores was performed according to Marques and Soares (2022). To examine the morphological characteristics of BS01, the samples were fixed in Karnovsky solution consisted of 2% (v/v) paraformaldehyde and 2.5% (v/v) glutaraldehyde prepared in 0.1 M sodium cacodylate buffer at pH 7.2–7.4 (Marques and Soares, 2022). Samples were then dehydrated in series of ethyl alcohol (10, 30, 50, 70, 90, and 100%), critical point-dried using CO_2_, mounted on aluminum stubs using double-sided carbon tape and coated with a gold film. The samples were subsequently analyzed by specific scanning with a JEOL IT 300 SEM at a voltage of 20 kV.

### 2.7 Carbohydrate and polyol analysis by high-performance liquid chromatography (HPLC)

The quantification of carbohydrates and polyols in BS01 and BS03 was carried out using HPLC. For sample preparation, 1 mL of water was added to 50 mg of each sample, which was then homogenized by sonication with 10 pulses at 20% amplitude for 15 s each, with 15 s intervals (Fisherbrand™ F505 Ultrasonic Dismembrator Cell Disruptor Cl-334 Converter, Thermo Fisher Scientific, Massachusetts, USA). The samples were centrifuged at 10,000 rpm for 15 min at 4 °C, and the resulting supernatant was filtered through a 0.45 μm membrane for HPLC analysis. Chromatographic analyses were performed on a Shimadzu LC- 20AT system using 0.1 mM NaOH as the mobile phase at a flow rate of 0.5 mL min^−1^. Analytes were separated on a C-611 Supelcogel column (30 cm × 7.8 mm) maintained at 60 °C, with a refractive index detector. Calibration curves for trehalose, glucose, mannitol, and glycerol were generated in water over the concentration range of 100–5,000 μg mL^−1^.

### 2.8 Confocal Raman microscopy

To investigate storage-induced damage in air-dried blastospores at the molecular level, we employed Raman spectroscopy to obtain surface spectra of fresh and dried *M. anisopliae* blastospores (BS01) after 0, 30, 60, and 90 days of storage at 28 ± 2 °C. Blastospores were produced as described in the fungal cultivation section. Following cultivation, microscope slides were prepared to assess cell integrity and verify the absence of contaminants. Cultures were then filtered through 10–12 μm filter paper to remove mycelial fragments and subsequently centrifuged at 5000 rpm for 10 min at 20 °C. The supernatant was discarded, and the pellet was washed three times with PBS (pH 7.4, 20 °C). The pellet was then air-dried in a laminar flow cabinet for 18 h, gently ground with a pestle, and further dried for 3 h, reaching an averagea_w_ = 0.121. The dried blastospores were aliquoted into 0.1 g portions, sealed in 5 × 5 cm aluminum foil sachets (moisture- and oxygen-impermeable), and stored at 28 ± 2 °C. Raman spectra were recorded from fresh washed cells and immediately after drying (time 0) and after 30, 60, and 90 days of storage.

Raman measurements were performed on samples that were initially removed from their pouch and subsequently localized under wide-field illumination. A 532 nm laser with an excitation power of 10 mW was employed. Spectra were acquired with a 100 × objective and a spot size of ~ 0.72 μm. The integration time for each acquisition was 2 s, with 10 accumulations. Spectral pre-processing was conducted using an in-house written script and included truncation to the fingerprint region (400–1800 cm^−1^), smoothing, baseline correction, and normalization. Raman microscopy was performed exclusively on the BS01 treatment due to the substantial labor requirements of the method. BS01 was selected as it exhibited the greatest reduction in viability after dehydration, rendering it the most pertinent condition for examining stress-induced cellular changes.

### 2.9 Primary metabolites analysis

In the present study, metabolomic analysis was intentionally restricted to the comparison between fresh and dried blastospores in order to characterize the primary metabolic shifts associated with dehydration, whereas storage-associated changes were followed using a targeted analysis of selected carbohydrates and polyols relevant to desiccation tolerance and viability maintenance.

To characterize the major shifts in primary metabolites between fresh and dried blastospores, metabolites were extracted and analyzed using gas chromatography–mass spectrometry (GC–MS), which enabled the detection of approximately 100 compounds, including sugars, organic acids, sugar alcohols, polyamines, and amino acids. Blastospores were cultivated as described in the fungal cultivation section, and microscope slides were prepared to verify cell integrity and the absence of contaminants. Twelve independent flasks were prepared, each treated as a biological replicate and processed individually. The fungal cultures were previously filtered through 10–12 μm filter paper to remove mycelium and subsequently centrifuged at 5000 rpm for 10 min at 4 °C. The supernatant was discarded, and the pellet was washed twice with PBS (pH 7.4, 4 °C), followed by a final wash with sterile distilled water (4 °C). The fresh pellets from six flasks were rapidly quenched in liquid nitrogen until further processing. Pellets from the remaining six flasks were dried in a laminar flow cabinet for 18 h, gently ground with a pestle, and subsequently dried for an additional 3 h, at which point the average water activity reached a_w_ = 0.121. Within the laminar flow cabinet, the temperature was maintained between 20 °C and 25 °C.

After drying, 0.1 g of each sample was collected to assess viability using the CFU method. This procedure was conducted solely to confirm sample integrity and was not intended as a quantitative assessment related to the metabolomic analysis itself. The dried pellets were then quenched in liquid nitrogen and freeze-dried overnight. For metabolite extraction, 5 mg of dried biomass were combined with 0.5 g zirconia/silica beads (0.5 mm diameter) and 1 mL of 80% MeOH containing ribitol (10 μmol L^−1^) as an internal standard. Biomass disruption was performed in a homogenizer at 6200 min^−1^ for 3 × 60 s. The resulting lysate was centrifuged at 19,000 g for 5 min, and 650 μL of the supernatant was transferred to 1 mL microreaction vessels. In parallel, a vessel containing 1 mL of 80% MeOH/10 μM ribitol was processed as a blank. Solvent evaporation was performed at 37 °C under a nitrogen gas stream for 80 min (Reacti-Therm heating and stirring module, Thermo Fisher Scientific Inc., MA, USA). Derivatization of dried extracts was carried out by adding 75 μL of methoxyamine (20 mg mL^−1^ in pyridine) for 90 min at 37 °C. Subsequently, 75 μL of N-methyl-N-(trimethylsilyl) trifluoroacetamide (MSTFA) was added for a second derivatization step, followed by incubation for 30 min. Finally, the derivatization mixture was centrifuged at room temperature for 5 min at 4000 rpm before being loaded into the GC–MS autosampler. A TSQ 9000 Triple Quadrupole GC–MS/MS (Thermo Electron, Dreieich, Germany) was used with an OPTIMA 5MS-0.25 μM, 30 m*0.25 μM column (Macherey-Nagel, Germany). Samples were processed with an injection of 1 μL sample at 280 °C with an ion source at 280 °C, a helium flow of 1 mL/min, splitless mode, and a temperature gradient of 80 °C for 3 min followed by an increase of 5 °C/min to 320 °C and a plateau for 2 min. The system was equilibrated for 2 min at 80 °C after each analysis. Mass spectra were recorded at 1 scan/s with a scanning range of 50–750 m/z. Pre-processing was performed on Xcalibur (V.4.5, Thermo Electron, Dreieich, Germany). Identification was performed by comparing mass spectra and retention times to an in-house reference database. Peak areas of compounds were normalised by the peak area of the internal standard ribitol and sample dry weigh.

### 2.10 Statistical analysis

All experiments were conducted using independent biological replicates as described in each section. Data are presented as mean ± standard error (SE), unless otherwise stated. Two fully independent experiments were performed for flow cytometry analyses, including separate fungal cultivation, blastospore production, and drying. Statistical analyses were performed using SigmaPlot (version 16, Systat Software Inc., San Jose, CA, USA). Viability data (CFU g^−1^) were log₁₀-transformed prior to analysis to ensure homoscedasticity and approximate normal distribution. Differences between the two treatments at each time point were assessed using Student’s t-test. Viability decay curves for BS01 and BS03 were fitted using a three-parameter logistic model.

The relationship between blastospore viability and protein carbonylation levels was evaluated using Spearman’s rank correlation analysis. The Spearman correlation coefficient (*ρ*) and corresponding *p*-values were calculated to determine the strength and significance of monotonic associations. Statistical significance was set at *p* < 0.05.

For metabolomic data (GC–MS), peak areas were normalized to the internal standard (norleucine) and to sample dry weight before analysis. Data processing, normalization, scaling, and multivariate statistical analyses were performed using MetaboAnalyst 4.0.^1^ After confirming normal distribution, unsupervised Principal Component Analysis (PCA) was applied to explore intrinsic clustering patterns and detect potential outliers. Supervised Partial Least Squares–Discriminant Analysis (PLS-DA) was subsequently conducted to identify metabolites contributing to group discrimination. The importance of metabolites in the PLS-DA model was assessed using Variable Importance in Projection (VIP) scores, calculated as the weighted sum of squared PLS loadings. Metabolites with higher VIP scores were considered more influential in distinguishing experimental groups.

Raman spectral preprocessing, including baseline correction, smoothing, normalization, and truncation to the fingerprint region (400–1,800 cm^−1^), was performed. Approximately 30 spectra were collected per sample from randomly selected regions. Mean spectra and corresponding standard deviations were calculated for each treatment and storage time to evaluate spectral variation.

Carbohydrate and polyol quantification by HPLC was performed using calibration curves (100–5,000 μg mL^−1^), and metabolite concentrations were determined by interpolation from linear regression models of peak area versus concentration.

### 2.11 AI Assistance in graphical abstract preparation

The graphical abstract for this manuscript was created with partial support from generative artificial intelligence (GenAI) tools, specifically ChatGPT 5.2. The tool was employed to assist in the development and refinement of visual components as well as accompanying descriptive text. All AI-assisted content was carefully reviewed and revised by the authors, who assume full responsibility for the final version presented.

## 3 Results

### 3.1 Viability of dried blastospores

Blastospores produced in a bioreactor and subsequently washed with PBS yielded 2.83 × 10^8^ blastospores mL^−1^ and had 96 ± 2% viability. After drying in a continuous airflow chamber, the blastospores with a water activity a_w_ = 0.335 ± 0.015 (BS03) had a viability of 1.15 ± 2.00 × 10^10^ CFU g^−1^, while those at a_w_ = 0.12 ± 0.018 (BS01) showed a viability of 1.18 ± 4.87 × 10^10^ CFU g^−1^. At the initial time point, corresponding to immediately after drying, no statistically significant differences were observed between treatments BS01 and BS03 (*p* = 0.927), indicating that extreme drying did not immediately affect viability. However, viability displayed temporal variation between the two treatments.

**Figure 1:**
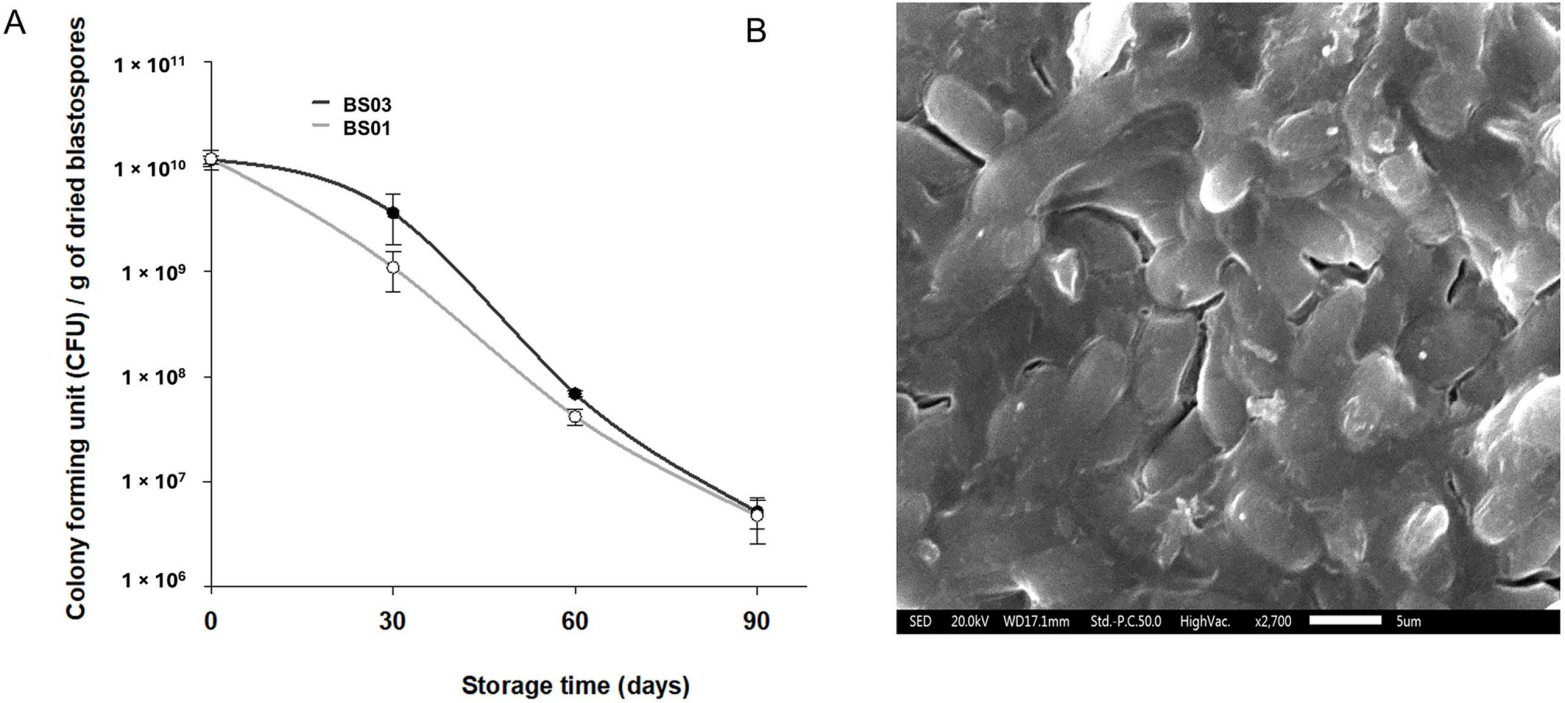
Colony-forming units (CFU) g^−1^ of *Metarhizium anisopliae* ESALQ1184 blastospores air-dried to water activities of 0.1 (BS01) and 0.3 (BS03) and stored at 28 ± 2 °C for 90 days **(A)**. Scanning electron micrograph of blastospores dried to a water activity a_w_ = 0.1 **(B)**. Error bars represent the standard errors of two independent experiments. Fresh blastospores, before drying, had 96 ± 2% viability.

Viability decay curves for BS01 and BS03 were fitted using a three-parameter logistic model (Table 1). The analysis revealed that water activity had a significant influence on blastospore viability (LR = 8.225, df = 3, *p* = 0.041). After 30 days of storage, a significant difference was detected between treatments (*p* = 0.027), with blastospores dried to a_w_ = 0.1 showing a viability of 1.10 ± 0.92 × 10^9^ blastospores g^−1^ of biomass, compared with 3.71 ± 2.66 × 10^9^ blastospores g^−1^ for those dried to a_w_ = 0.3 (Figure 1A). By 60 days of storage, however, no significant differences in viability were detected between treatments (*p* > 0.05). These results indicate that extreme drying accelerates viability loss in the first month, but long-term loss is similar for both treatments. Scanning electron microscopy of BS01 biomass further revealed blastospores embedded in a mucilage-like matrix (Figure 1B). This matrix consists of fibrils that coat the relatively thin cell wall and also extend beyond the blastospores. Additionally, this substance is insoluble, as reported by Alkhaibari et al. (2016).

**Table 1:** Parameters of the nonlinear logistic models fitted to the colony-forming unit data of *Metarhizium anisopliae* ESALQ 1184 blastospores dried in an airflow chamber to two water activity levels, a_w_ = 0.1 (BS01) and a_w_ = 0.3 (BS03), and vacuum-stored at 28 ± 2 °C for 90 days.

Treatment	Water activity (mean ± SE)	Model parameters			Adjusted *R^2^*
		*a*	*b*	*x0*	
BS01	0.126 ± 0.018	1.179 × 10^10^	4.865	18.775	0.818
BS03	0.335 ± 0.015	1.155 × 10^10^	6.250	26.439	0.853

### 3.2 Effect of drying on blastospores’ plasma membrane permeability and reactive oxygen species production

**Figure 2:**
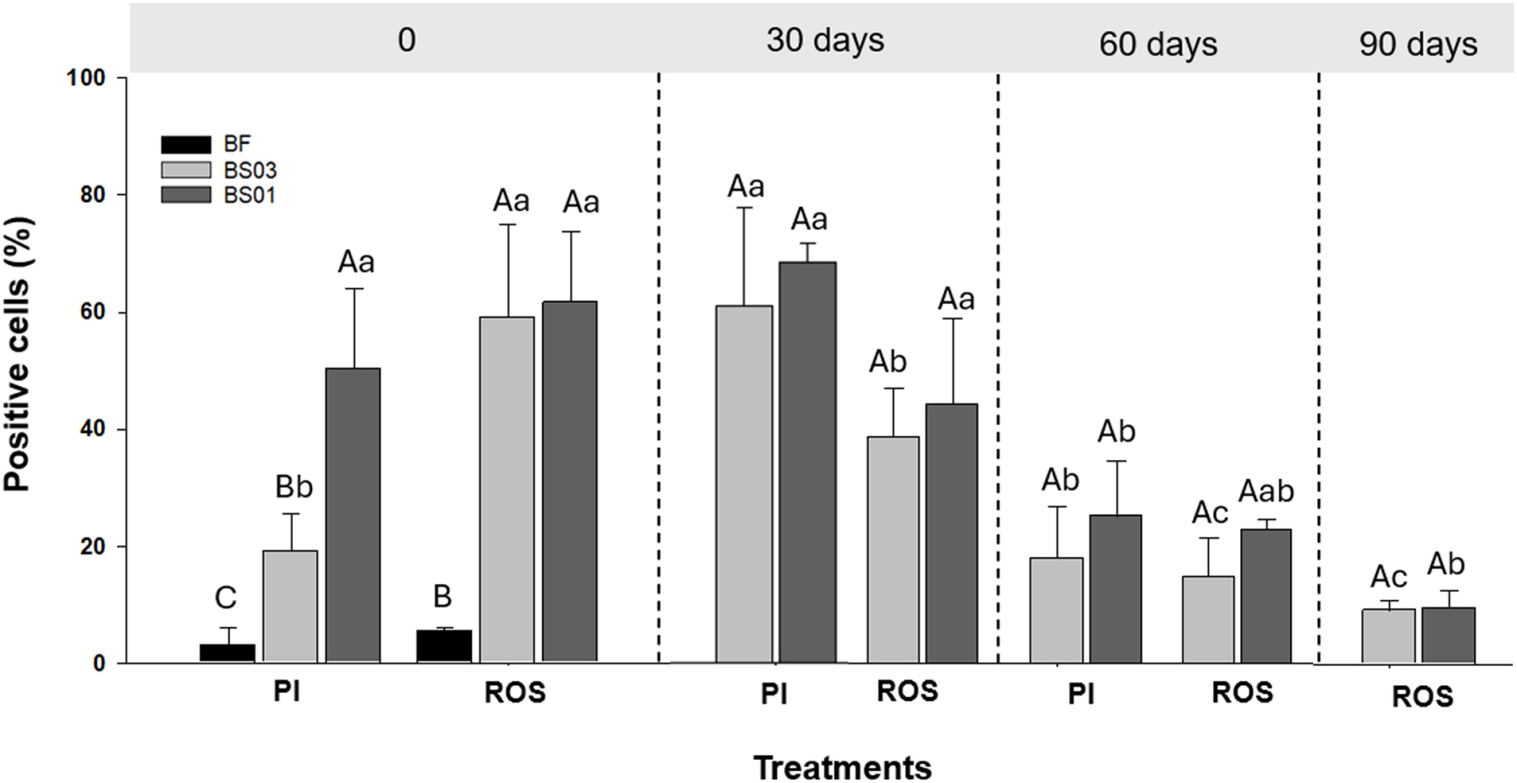
Percentage of fresh (BF) and air-dried *Metarhizium anisopliae* ESALQ1184 blastospores at water activity a_w_ = 0.3 (BS03) and 0.1 (BS01) labeled with propidium iodide (PI) and a fluorophore for reactive oxygen species (ROS) detection immediately after drying (time 0), and after 30, 60, and 90 days of vacuum storage at 28 ± 2 °C. Each value represents the mean of two independent experiments, and error bars indicate the standard error of the mean. Different uppercase letters denote statistically significant differences between treatments on the same assessment day, while different lowercase letters indicate statistically significant differences within the same treatment across days (significance level: *p* < 0.05).

The percentage of blastospores showing membrane damage fluctuated significantly over time for BS01 (*p* = 0.007) and BS03 (*p* = 0.002), as did the presence of ROS for BS01 (*p* = 0.014) and BS03 (*p* = 0.003) (Figure 2).

Blastospores subjected to drying exhibited pronounced plasma membrane damage, which was most severe under the extreme drying condition (BS01) at time 0 (Figure 2). In this treatment, the proportion of positive cells remained stable during the first 30 days of storage but declined after 60 days and ultimately reached zero by day 90. In contrast, for BS03, the proportion of positive cells immediately after drying (time 0) was significantly lower than in BS01 but higher than in non-dried blastospores (BF), indicating that moderate drying preserves plasma membrane integrity better than extreme drying. After 30 days of storage, the proportion of positive cells in BS03 increased threefold, from 20% at 0 to 61%, with no statistically significant difference compared with BS01 on the same day. Similar to BS01, however, the proportion of positive cells in BS03 decreased significantly after 60 days and dropped to zero by day 90 (Figure 2).

There were no significant differences in ROS levels between BS01 and BS03 at any time point. In both treatments, ROS levels declined over time and reached minimum values by the end of the 90-day storage period. However, at time 0, dried blastospores exhibited a substantially higher proportion of ROS-positive cells (60%) compared with fresh blastospores (BF, 8%) (Figure 2).

### 3.3 Lipid peroxidation

**Figure 3:**
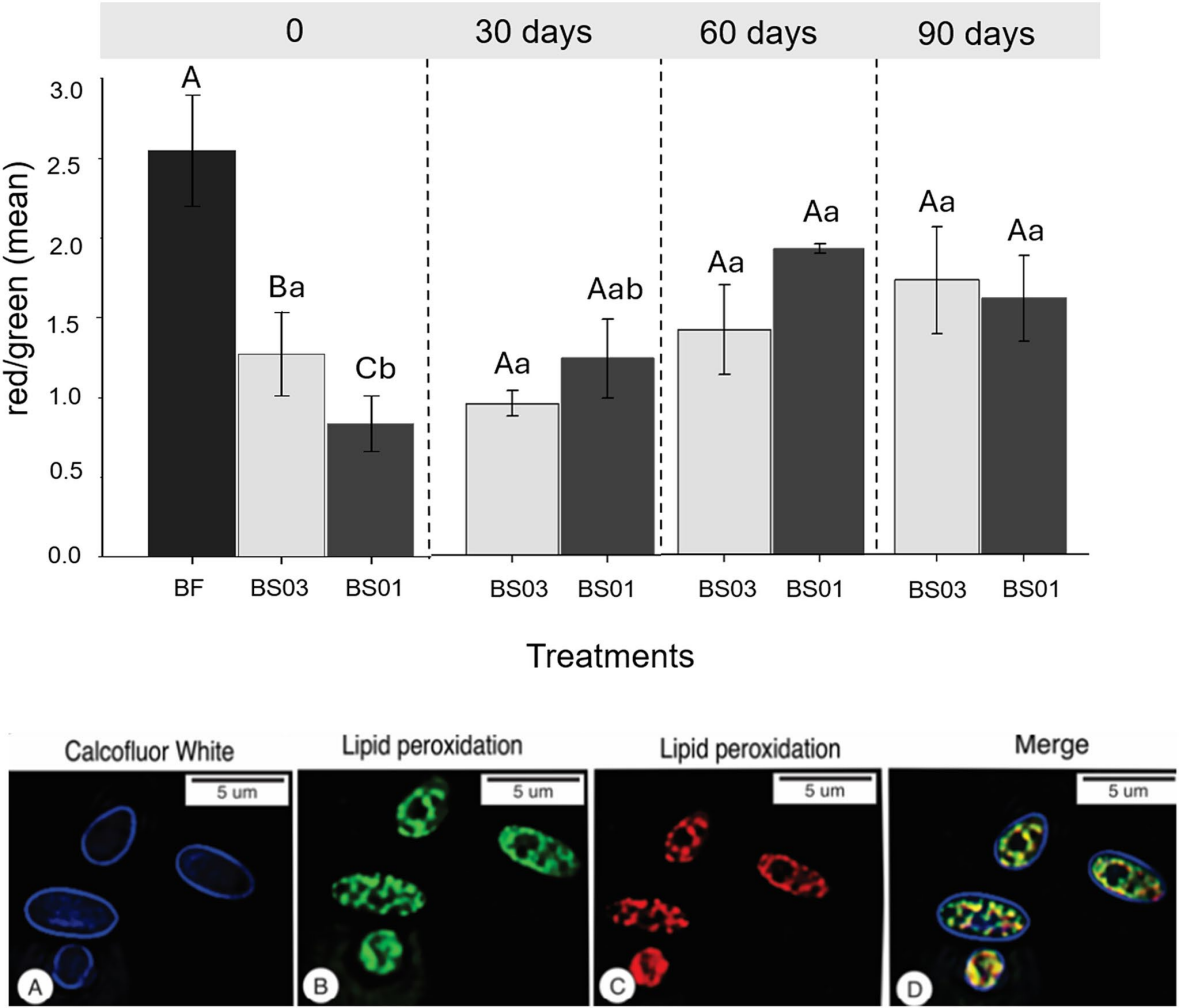
Lipid peroxidation indicated by red/green fluorescence ratio of BODIPY™ 581/591 C11 of fresh (BF) and air-dried *Metarhizium anisopliae* ESALQ1184 blastospores at water activity a_w_ = 0.1 (BS01) and a_w_ = 0.3 (BS03) immediately after drying (time 0), and after 30, 60, and 90 days of storage at 28 ± 2 °C in vacuum. The greater this ratio, the lesser the lipid peroxidation observed in the sample. Each value represents the mean of two independent experiments, and error bars indicate the standard error of the mean. Different uppercase letters denote statistically significant differences between treatments on the same assessment day, while different lowercase letters indicate statistically significant differences within the same treatment across days (significance level: *p* < 0.05). Analysis under fluorescent microscopy of air-dried blastospores at 0.1 a_w_ after 30 days of storage at 28 ± 2 °C. **(A–D)** Blastospores after calcofluor and lipid peroxidation. **(A)** Calcofluor-stained fungal cell wall (Blue); BD–Image-iT^®^ lipid peroxidation stains method. Note the lipid reduced (Red-**C**) and after peroxidation (Green-**B**). **D**–Merged image. Note the colocalization of the green and red channels.

Dried blastospores exhibited higher lipid peroxidation levels compared to fresh samples (*p* = 0.019), with BS01 showing significantly higher peroxidation than BS03 at time 0 (Figure 3). There was no significant increase in lipid peroxidation levels in BS03 over the storage period (*p* = 0.215), but a statistically significant reduction in lipid peroxidation was observed in BS01 when comparing 60 and 90 days to time 0 (*p* = 0.032). Fluorescence images illustrated an intense but non-homogeneous occurrence of lipid peroxidation in the blastospore membranes (BS01) after 30 days of storage (Figures 3A–D).

### 3.4 Protein carbonylation

**Figure 4:**
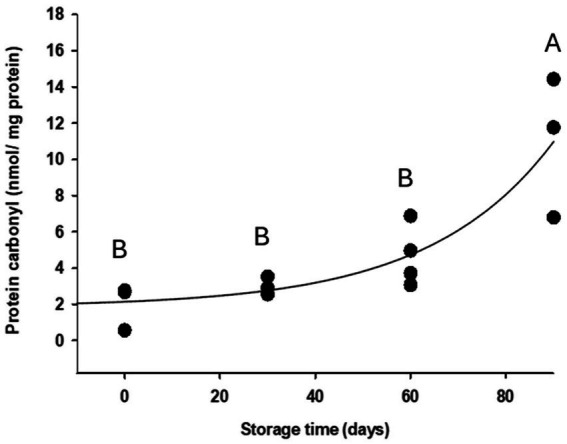
Protein carbonyl content (nmol/mg protein) in air-dried *Metarhizium anisopliae* ESALQ1184 blastospores at water activity a_w_ = 0.1 after drying (time 0), and after 30, 60, and 90 days of vacuum storage at 28 ± 2 °C. Different uppercase letters indicate statistically significant differences among treatments from two independent experiments (significance level: *p* < 0.05).

The progression of protein oxidation over storage time was evident in blastospores dried to an a_w_ = 0.1, as indicated by a time-dependent increase in protein carbonyl content (Figure 4). The data were fitted to a three-parameter exponential model described by the equation $f(x)=1.899+0.286\times 1.039^{x}(R^{2}=0.736)$. Immediately after drying, the carbonyl content was 2.04 ± 0.71 nmol mg^−1^ protein (Figure 4). This value increased significantly to 11.02 ± 2.23 nmol mg^−1^ protein after 90 days, representing an approximately fivefold increase. The most pronounced change occurred between days 60 and 90, when carbonyl levels rose from 4.69 ± 0.83 to 11.02 ± 2.23 nmol mg^−1^ per protein, corresponding to a 2.3-fold increase.

In addition, Spearman’s rank correlation revealed a strong and significant negative association between blastospore viability and protein carbonylation (*ρ* = −0.94, *p* < 0.0001). These findings demonstrate that higher protein carbonylation results in a steady decline in blastospore survival, highlighting the negative impact of oxidative damage to proteins on fungi during storage.

### 3.5 Sugar and polyol analysis

**Figure 5:**
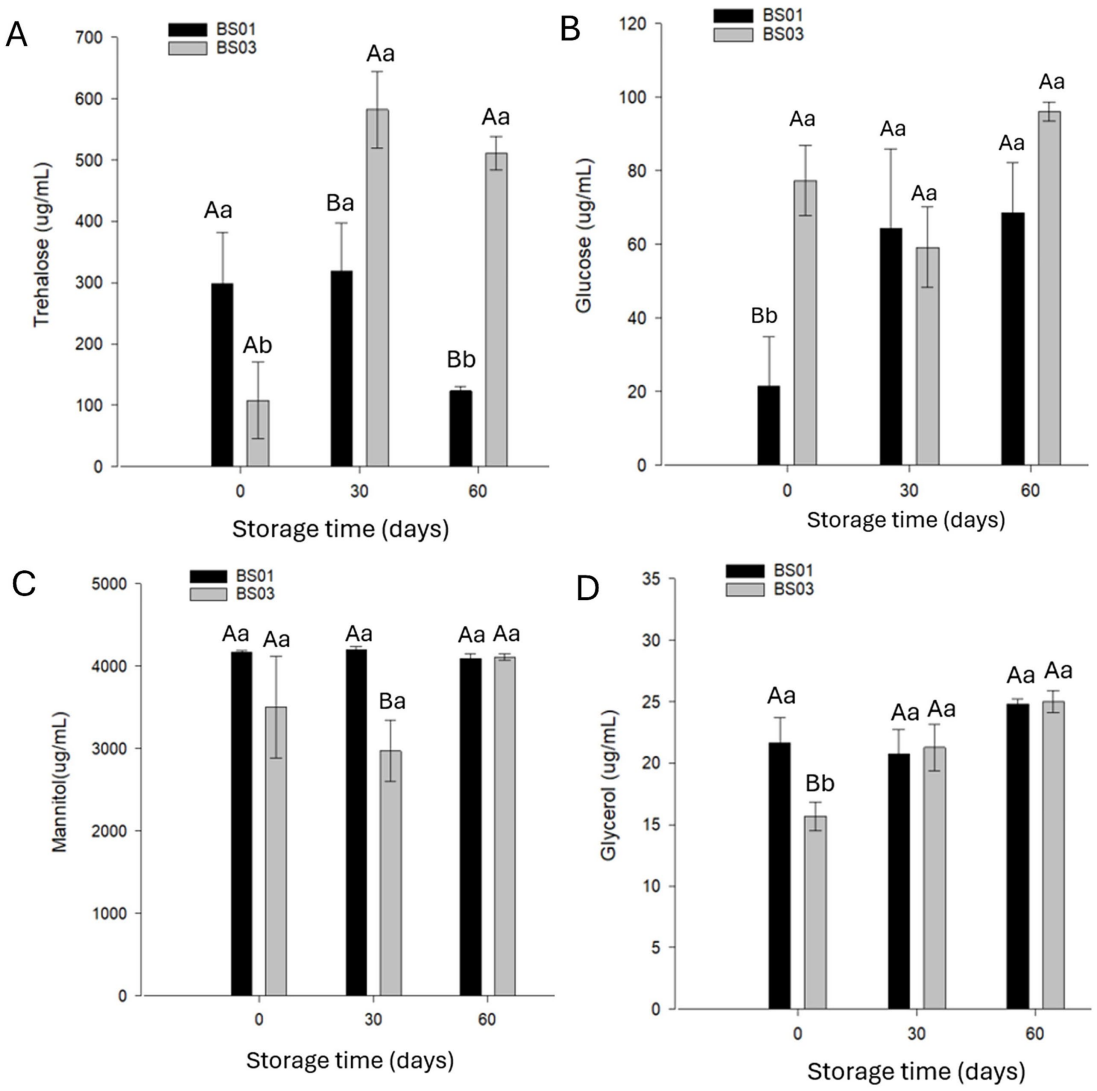
Sugar [Trehalose **(A)** and Glucose **(B)**] and polyol [Mannitol **(C)** and Glycerol **(D)**] concentrations in air-dried *Metarhizium anisopliae* ESALQ1184 blastospores at water activity a_w_ = 0.1 (BS01) and a_w_ = 0.3 (BS03) after drying (time 0), and after 30 and 60 days of vacuum storage at 28 ± 2 °C. Different uppercase letters denote statistically significant differences between treatments on the same assessment day, while different lowercase letters indicate statistically significant differences within the same treatment across days (significance level: p < 0.05). Data are means ± standard error from two independent experiments.

We analyzed the accumulation of trehalose and glucose, together with the polyols mannitol and glycerol, during storage (Figure 5). Different trends were observed for BS01 and BS03 in terms of trehalose and glucose concentrations. In BS01, trehalose levels remained stable up to 30 days of storage, after which a significant decline was detected between days 30 and 60, suggesting possible metabolic consumption of this sugar. In parallel, glucose levels showed a marked increase from day 0 to day 30, followed by stabilization between days 30 and 60. In contrast, BS03 exhibited a significant increase in trehalose concentration between day 0 and day 30, with stabilization thereafter, while glucose levels remained unchanged throughout storage. For mannitol and glycerol, no significant variation was observed in BS01. However, in BS03, glycerol levels increased significantly between day 0 and day 60.

### 3.6 Confocal Raman microscopy

**Figure 6:**
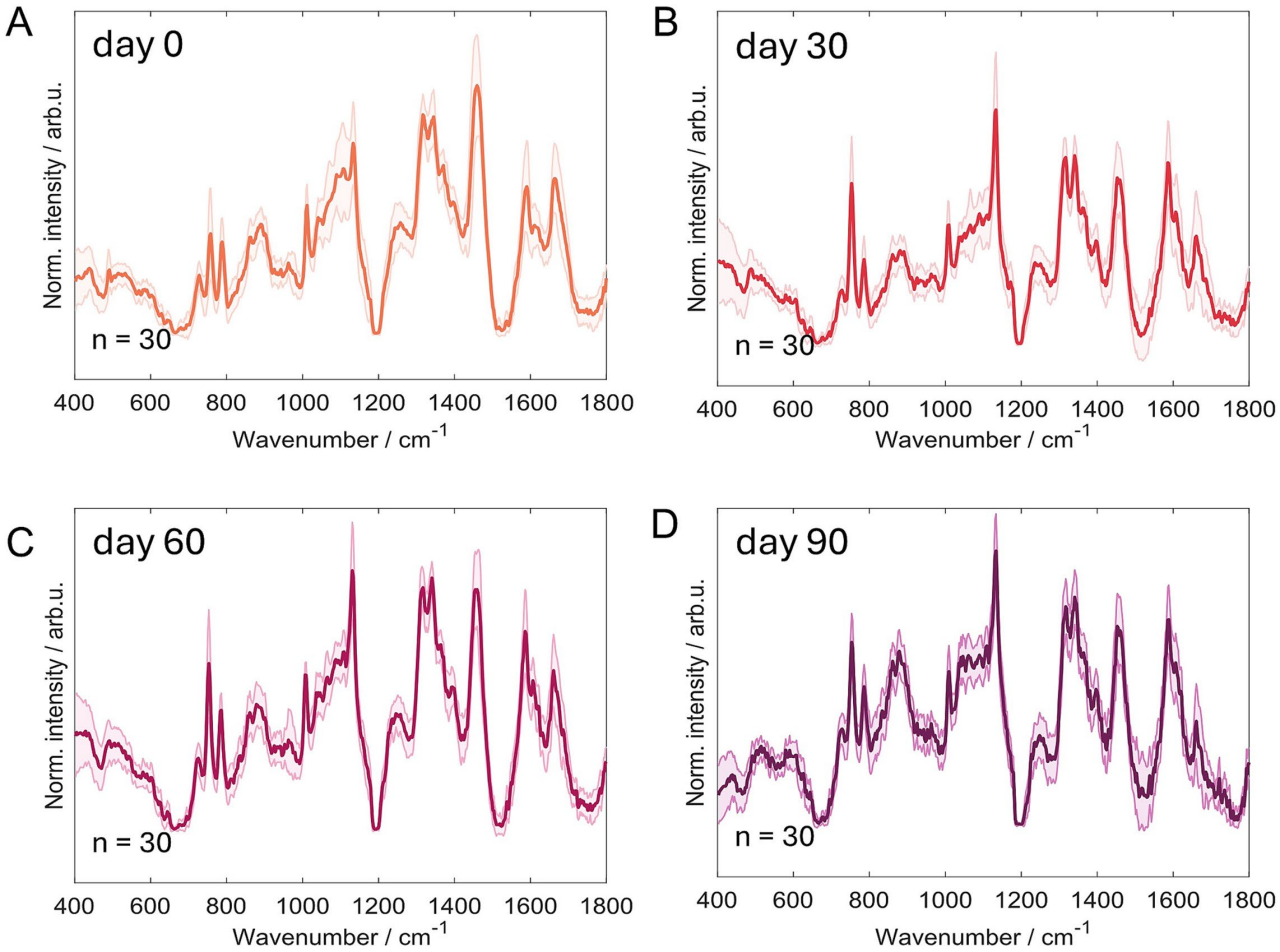
Mean Raman spectra ± standard deviation of air-dried *Metarhizium anisopliae* ESALQ1184 blastospores water activity a_w_ = 0.1 (BS01) after drying (time 0) **(A)**, and after 30 **(B)**, 60 **(C)**, and 90 **(D)** days of vacuum storage at 28 ± 2 °C.

Spectra for each dried blastospore (BS01) sampling day are shown in Figure 6, with the shaded region representing the standard deviation across spectra. The observed variation is modest, and the mean spectra accurately represent the samples (Figure 6). No outliers were detected.

**Figure 7:**
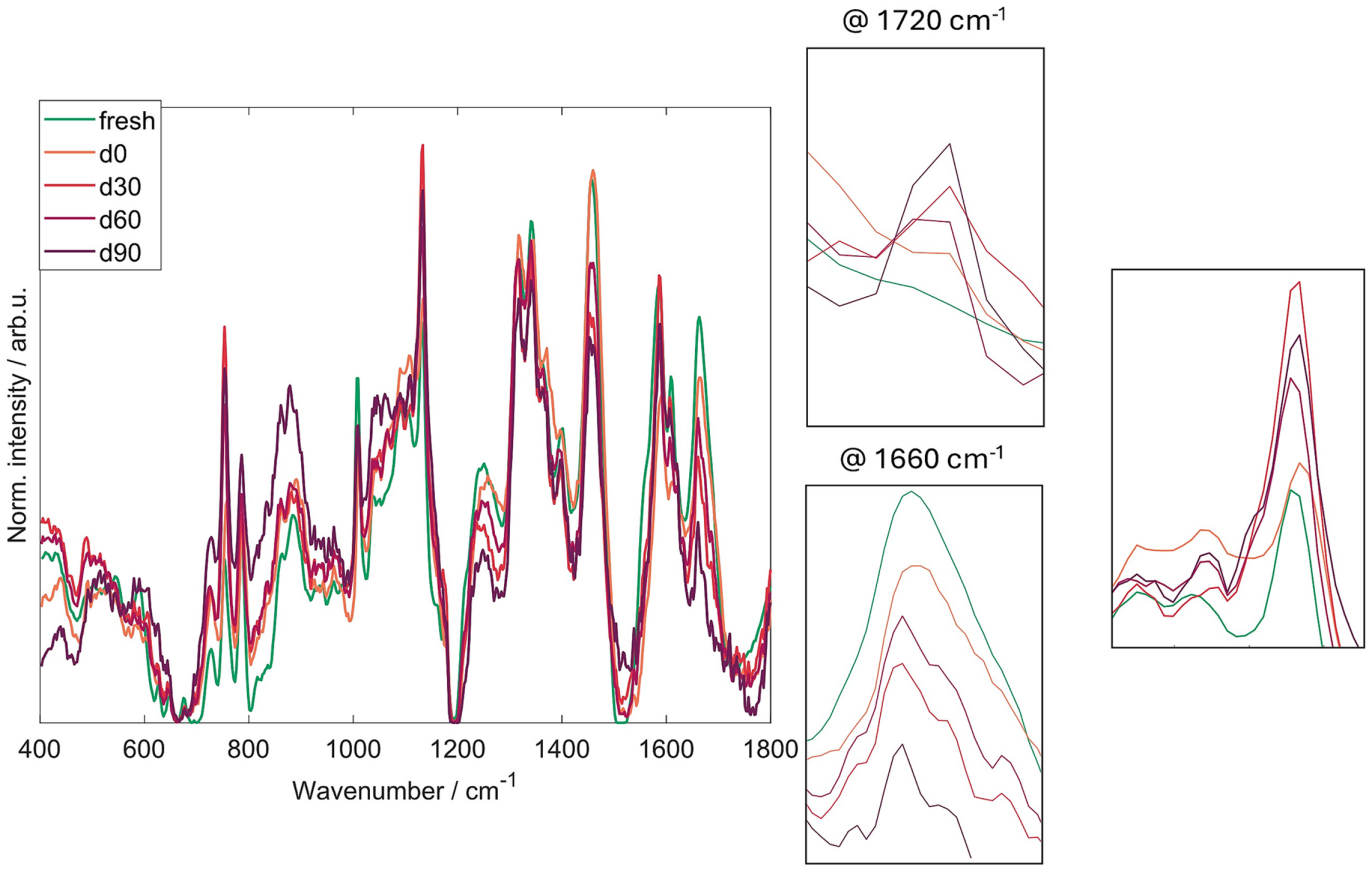
Normalized Raman spectra of air-dried *Metarhizium anisopliae* ESALQ1184 blastospores at water activity a_w_ = 0.1 (BS01) after drying (time 0), and after 30, 60, and 90 days of vacuum storage at 28 ± 2 °C.

Mean Raman spectra were collected from fresh samples, samples dried at time 0, and samples stored for 30, 60, and 90 days, and plotted together in Figure 7 to show how the sample’s molecular composition changes over time. Raman spectroscopy measures vibrations of chemical bonds, so changes in peak intensities or the appearance of new features indicate changes in the amounts or the chemical environment of different molecular components. Our initial interpretation was that the observed differences could be explained solely by concentration changes due to desiccation (drying, which can make remaining components appear more concentrated). However, significant differences persisted at 30 and 60 days, indicating true molecular alterations beyond simple concentration effects caused by drying.

The spectral region around 1720 cm^−1^ is associated mainly with carbonyl (C=O) vibrations found in carbonyl groups. This band increases with storage time and is not present in the fresh samples, which is consistent with protein carbonylation (a marker of protein oxidation) (Zhou et al., 2025; Giancaspro et al., 2022). The Amide I region around 1,660 cm^−1^ reports on protein secondary structure, for example, the balance between alpha-helix and beta-sheet content. We observed changes in the intensity of this region and the emergence of a shoulder relative to the fresh sample, which may reflect modifications in secondary structure or in the local protein environment, such as changes in hydration or packing around the proteins (Rygula et al., 2013).

**Figure 8:**
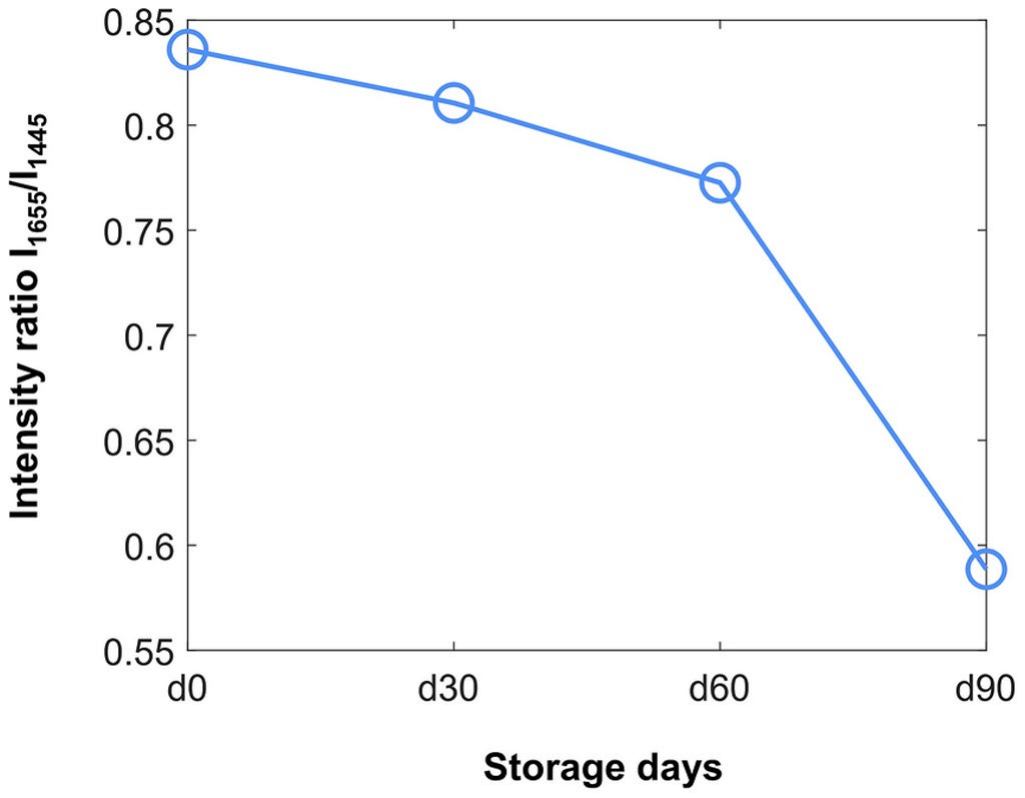
Lipid-unsaturation index derived from Raman spectroscopy of air-dried *Metarhizium anisopliae* ESALQ1184, defined as the intensity ratio between the peaks at 1655 cm^-1^ and 1445 cm^-1^. The ratio declines with storage time, reflecting an increase in lipid saturation over time. Data are means ± standard error from two independent experiments.

In addition, we quantified lipid unsaturation using the lipid-unsaturation index, defined as the intensity ratio I_1655_/I_1445_ (this ratio is a standard metric in Raman lipid analyses; see Figure 8). This ratio decreases with storage time, indicating that lipids are becoming more saturated (fewer double bonds) during storage, i.e., a progressive loss of lipid unsaturation (Kochan et al., 2014; Tratwal et al., 2022).

### 3.7 Primary metabolites of fresh and dried blastospores

**Figure 9:**
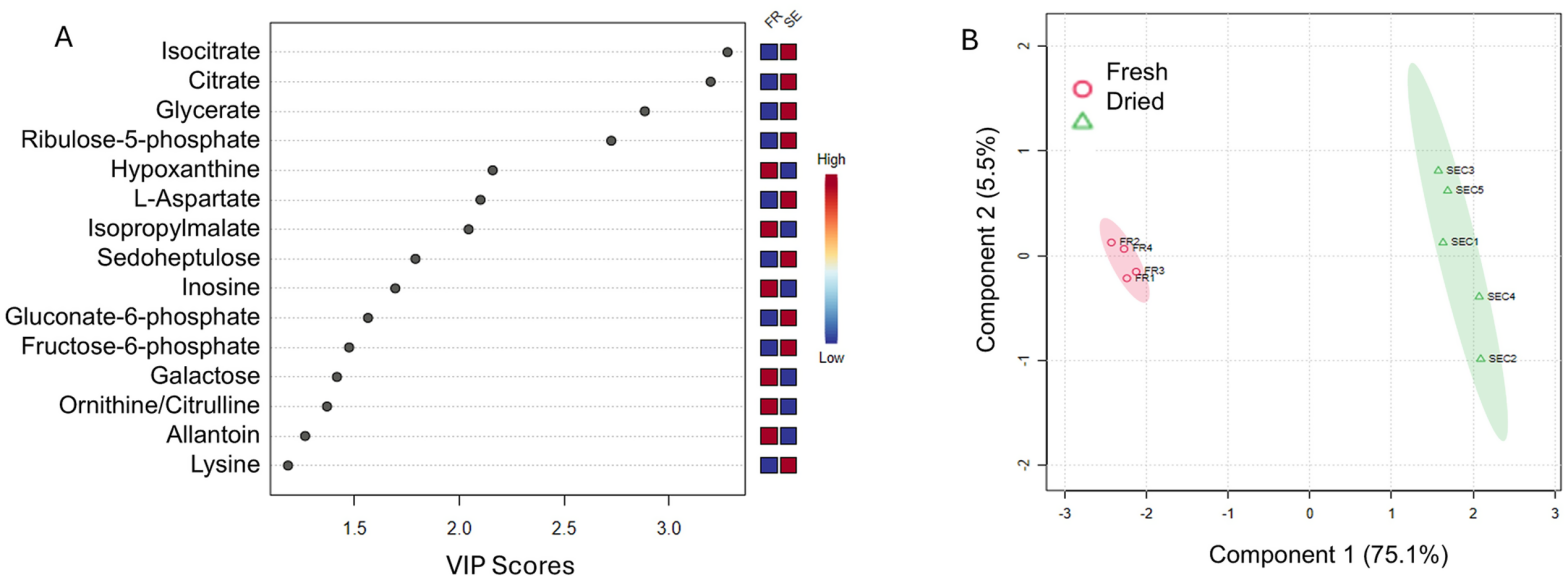
Analysis of the VIP (variable importance in projection) scores for primary metabolites of *M. anisopliae* ESALQ1184 blastospores. This plot identifies the metabolites that contribute most to distinguishing between fresh and dried samples. The horizontal cutoff line at VIP = 1 indicates the relevance threshold; metabolites with scores above this line are considered important. The bar colors indicate the relative abundance of each metabolite in the two conditions, with the scale ranging from blue (low) to red (high) **(A)**. Principal component analysis (PCA) of *Metarhizium anisopliae* ESALQ1184 blastospores before and after air-drying. Red circles represent fresh samples before drying, while green triangles represent air-dried blastospores to a_w_ = 0.1 **(B)**.

In dried blastospores, there is an accumulation of intracellular intermediates of the tricarboxylic acid (TCA) cycle, such as citrate and isocitrate, as well as glycolytic and pentose phosphate pathway intermediates, including fructose-6-phosphate, 6-phosphogluconate, ribulose-5-phosphate, and sedoheptulose (Figure 9B). This pattern suggests a partial arrest of catabolic pathways, resulting in the retention of energy-rich intermediates and the production of NADPH via the pentose phosphate pathway, which is crucial for counteracting oxidative stress induced by dehydration. Amino acids such as L-aspartate and lysine also show elevated concentrations (see Figure 9).

In contrast, fresh blastospores exhibit higher levels of metabolites associated with active metabolism, including nucleotides and their derivatives (inosine, hypoxanthine, allantoin), intermediates of amino acid biosynthesis (isopropylmalate), and metabolizable sugars (galactose) (Figure 9A). Amino acids such as ornithine and citrulline reflect biosynthetic activity and nitrogen processing. This profile indicates that, under hydrated conditions, cells maintain continuous energy metabolism, macromolecule biosynthesis, and energy production.

The principal component analysis (PCA) of the concentration of primary metabolites separates fresh and dried blastospore samples into two distinct clusters along the first principal component (C1), which explains 75.1% of the total variance. Fresh samples (FR1–FR4) cluster on the negative side of C1, whereas dried samples (SEC1–SEC5) cluster on the positive side. This clear separation indicates a metabolic distinction between hydrated and dehydrated blastospores (Figure 9B). The data obtained from the metabolome analysis can be found in the supplementary material (Supplementary Table S1).

## 4 Discussion

The practical application of blastospores in biological pest control requires preserving their viability through a dehydration (desiccation) process to extend shelf life. In this study, we investigated the cellular mechanisms underlying the loss of viability of *M. anisopliae* ESALQ1804 blastospores subjected to airflow drying and stored at 28 ± 2 °C, using a combined approach of biochemical assays, Raman microscopy, and metabolomic profiling over a 90-day period. Our results show that the loss of viability is intrinsically linked to oxidative stress, manifested by damage to the plasma membrane, lipids, and proteins. Additionally, drying promotes the accumulation of free amino acids and intermediates of the respiratory and TCA cycle pathways.

### 4.1 Immediate dehydration damage and membrane integrity

Although blastospores dried to different water activities (BS01 and BS03) exhibited comparable viability immediately after drying, molecular damage differed significantly. At time 0, BS01 showed a higher proportion of PI-positive cells and significantly elevated levels of lipid peroxidation compared to BS03. Lipid peroxidation, a hallmark of oxidative stress, is driven by reactive oxygen species (ROS), which we also detected at high levels in the dried cells. This process constitutes a chain reaction in which ROS attack polyunsaturated fatty acids in cellular membranes, generating lipid radicals that react with molecular oxygen to form peroxyl radicals, propagating damage by abstracting hydrogen atoms from neighboring lipid molecules (Valgimigli, 2023). This propagation cycle amplifies membrane damage, compromising its integrity and function, a phenomenon previously reported in dried *Saccharomyces cerevisiae* (Garre et al., 2010) and other anhydrobiotic organisms (Hermes-Lima and Zenteno-Savin, 2002).

The higher lipid peroxidation in BS01 immediately after drying suggests that the oxidative stress induced by more intense dehydration caused sublethal injuries. Although these injuries did not compromise short-term viability, they progressively impaired cellular survival during storage, consistent with the reduced viability of BS01 compared to BS03 after 30 days of storage. Furthermore, rehydration plays a crucial role in viability loss. Dehydration increases phospholipid headgroup packing, thereby enhancing van der Waals interactions and raising the phase transition temperature (Tm), which causes lipids to enter a gel phase (Crowe et al., 1992). Rapid rehydration can trigger abrupt phase transitions, resulting in micro-ruptures that compromise membrane integrity (Laroche and Gervais, 2003). Therefore, we hypothesize that the reduced viability of BS01 after 30 days is due to a combination of more severe membrane damage and rehydration shock.

Notably, the late decline in lipid peroxidation observed in the BS01 group does not necessarily indicate recovery. This reduction may be associated with both substrate depletion and metabolic arrest. As oxidative damage progresses, polyunsaturated fatty acids, the primary substrates for lipid peroxidation, may become progressively depleted, limiting the continuation of peroxidative reactions. At the same time, the pronounced loss of viability suggests substantial metabolic impairment, which may reduce ROS generation and consequently decrease lipid peroxidation levels. Because residual lipid content and metabolic activity were not directly measured at the corresponding time points, it is not possible to determine which mechanism predominates. Thus, the late reduction in lipid peroxidation likely reflects a combination of substrate exhaustion and metabolic collapse rather than an adaptive protective response.

Compared to fresh cells, both BS01 and BS03 showed higher proportions of PI-positive cells, elevated ROS levels, and increased lipid peroxidation. ROS production occurs under various stress conditions, including yeast dehydration (Dupont et al., 2014), mechanical damage in *Glomus intraradices* mycelium (Fester and Hause et al., 2005), and in seeds (Bailly, 2004; Bailly et al., 2008), as well as during cell rehydration (Catalá et al., 2010). Cells employ enzymatic and non-enzymatic mechanisms to regulate ROS (Sies, 1991). Under physiological conditions, reactive oxygen species (ROS), including superoxide anion (O₂•-), hydrogen peroxide (H₂O₂), and hydroxyl radical (HO•), are continuously produced and neutralized. Oxidative stress arises when ROS production exceeds the cell’s capacity for detoxification. Although we cannot distinguish whether the ROS detected were produced during rehydration or accumulated during drying, or both, we can confirm their presence, indicating that the blastospores were experiencing oxidative stress. Notably, Iwanicki et al. (2020a, 2020b) observed a stronger oxidative stress response in blastospores compared to hyphae of *M. anisopliae*, and higher TCA cycle and respiration activity in blastospores may also contribute to increased ROS production.

### 4.2 Progressive deterioration and accumulation of oxidative damage

During storage (60 and 90 days), the proportion of PI-positive cells, ROS, and lipid peroxidation markers declined sharply, while viability dropped to <1 and 0.1%, respectively. This decrease does not indicate recovery but rather cumulative deterioration. Reduced PI staining may result from DNA fragmentation or loss of intracellular content, limiting dye accessibility. Similarly, the decline in ROS and lipid peroxidation detection is attributed to the metabolic inactivation and degradation of the markers themselves. Reactive aldehydes react with proteins or DNA, while membrane degradation and depletion of unsaturated lipids reduce the available substrates (Moldogazieva et al., 2023).

In contrast, we observed a progressive and significant increase in protein carbonylation, indicative of oxidative protein damage, reaching 11.02 ± 2.23 nmol mg^−1^ protein after 90 days. For comparison, Lushchak et al. (2009) reported protein carbonyl levels ranging from 2.5 to 6.2 nmol mg^−1^ protein in *S. cerevisiae* treated with various concentrations of menadione, an oxidative stress inducer, which are similar to the values we observed at 0, 30, and 60 days. Cabiscol et al. (2000) analyzed proteins oxidatively damaged in *S. cerevisiae* under stress conditions and detected carbonyl groups generated by hydrogen peroxide or menadione in aerobically respiring cells. Under these stress conditions, fermenting cells exhibited lower viability than aerobically respiring cells, accompanied by increased peroxide generation and a higher content of protein carbonyls and lipid peroxides. However, even under these high-stress conditions, the maximal carbonyl concentration reported was 1.82 nmol mg^−1^ protein, markedly lower than the levels we observed in air-dried blastospores after 90 days of storage.

Carbonylation is an irreversible process primarily driven by ROS (Kalemba and Pukacka, 2014). Dehydration promotes protein denaturation, aggregation, and loss of activity (Prestrelski et al., 1993; Potts, 1994). Protein carbonylation correlates with reduced cell viability, as demonstrated in *S. cerevisiae* (França et al., 2005, 2007). Mechanistically, carbonylation can inactivate key metabolic enzymes involved in glycolysis, the tricarboxylic acid cycle, and ATP synthesis, thereby compromising cellular energy homeostasis (England and Cotter, 2004). It may also impair antioxidant defense systems and disrupt redox-sensitive signaling pathways essential for stress responses and cellular repair (Curtis et al., 2012). In addition, carbonylated proteins are prone to aggregation and may overwhelm proteostasis networks, including the proteasome and chaperone systems, further exacerbating cellular dysfunction (Dalle-Donne et al., 2006). Therefore, the exponential increase in carbonyl levels observed at 90 days reflects intense oxidative protein damage in the blastospores, resulting in low viability.

Correlations between Raman spectral changes and independent assays support storage- and desiccation-related molecular changes in blastospores, yielding spectral signatures that reflect these biochemical transitions over time, including increases in carbonyl groups and in lipid saturation. The Raman measurements are straightforward and non-destructive, requiring no sample preparation, with a typical measurement session spanning about 1 h for multiple spectra across regions (Jensen et al., 2024). The rapid throughput and the ability to monitor subtle spectral changes with a single analytical method support the continued development of Raman-based approaches and the construction of a spectral library to guide future studies.

### 4.3 Cellular protection mechanisms and residual metabolic activity

Organisms withstand dehydration by accumulating compatible solutes such as trehalose and polyols, which stabilize membranes and proteins and mitigate oxidative damage (Crowe et al., 1987, 1992, 1998; Welsh, 2000; Benaroudj et al., 2001; Tapia and Koshland, 2014; Kuczyńska-Wiśnik et al., 2024; Hallsworth and Magan, 1994a, 1994b). In our study, BS01 exhibited a progressive decline in trehalose during storage, accompanied by increased glucose levels at 30 and 60 days, which we interpret as a possible indication of trehalose mobilization and progressive loss of protective capacity. Although metabolic activity is expected to be minimal at very low water activity, we consider that residual metabolic processes and/or gradual mobilization of intracellular reserves may still contribute to this pattern. Such depletion may exacerbate oxidative damage by reducing membrane and protein stabilization. In contrast, BS03 showed increased trehalose after 30 days, indicating residual metabolic activity at a_w_ = 0.3, which may have temporarily enhanced structural protection and contributed to its greater stability.

Metabolomic analysis further revealed accumulation of TCA cycle and glycolytic/pentose phosphate intermediates in BS01, consistent with metabolic imbalance and partial catabolic arrest. The increased levels of amino acids, including L-aspartate and lysine, likely reflect stress-induced proteolysis and disruption of anabolic pathways rather than adaptive protection. Although amino acid accumulation has been associated with osmotic and oxidative stress responses (Wang et al., 2024; Batista-Silva et al., 2019; Han et al., 2021), in the context of declining trehalose levels and progressive protein carbonylation, these changes are more consistent with metabolic dysregulation. Although enzymatic activities and metabolic fluxes were not directly measured, the combined metabolomic, biochemical, and spectroscopic data are consistent with progressive metabolic imbalance and partial catabolic arrest during blastospore viability loss.

### 4.4 Conclusion and practical implications

Storage under vacuum and low water activity did not prevent oxidation of cellular components, revealing residual oxygen and the need for improved strategies, including oxygen absorbers, to extend shelf life (Iwanicki et al., 2021; Faria et al., 2010). The core finding that the long-term loss of viability in *M. anisopliae* blastospores is primarily driven by cumulative oxidative damage, particularly irreversible protein carbonylation, in conjunction with the depletion of endogenous trehalose, carries critical implications for biopesticide industrial development. Future formulation strategies should move beyond simple carrier systems toward multifunctional matrices incorporating potent antioxidants capable of mitigating both lipid peroxidation and protein oxidation. Process optimization is equally essential; precise control of the drying phase is crucial, as industrial protocols should aim for a water activity level that minimizes sublethal oxidative stress. When dehydration exceeds the tolerance limits of the cells, macromolecular degradation is accelerated due to irreversible conformational changes and oxidation reactions (Potts, 1994). Moreover, supplementation with exogenous xeroprotectants (e.g., trehalose or polyols) is required to compensate for the limited endogenous stress response. Finally, the demonstrated capacity of Raman spectroscopy to non-destructively detect molecular degradation signatures (e.g., lipid saturation indices) represents a significant technological advance for real-time quality control, enabling the early identification of potentially unstable batches before detectable losses in viability occur.

This study was the first comprehensive attempt to combine biochemical analysis, Raman microscopy, and metabolomic profiling to investigate the mechanisms of viability loss in entomopathogenic fungal blastospores following drying and during storage. This synergistic approach provided a detailed molecular understanding, revealing that long-term shelf-life failure is primarily driven by cumulative oxidative damage, specifically, irreversible protein carbonylation concurrent with the depletion of endogenous trehalose.

## Data Availability

The raw data supporting the conclusions of this article will be made available by the authors, without undue reservation.

## Footnotes

1 https://www.metaboanalyst.ca
